# A Review of Recent Advances and Future Directions in the Management of Salinity Stress in Finger Millet

**DOI:** 10.3389/fpls.2021.734798

**Published:** 2021-09-16

**Authors:** Wilton Mbinda, Asunta Mukami

**Affiliations:** ^1^Department of Biochemistry and Biotechnology, Pwani University, Kilifi, Kenya; ^2^Pwani University Biosciences Research Centre (PUBReC), Pwani University, Kilifi, Kenya; ^3^Department of Life Sciences, South Eastern Kenya University, Kitui, Kenya

**Keywords:** climate change, breeding, finger millet, salinity stress, secondary metabolites

## Abstract

Salinity stress is a major environmental impediment affecting the growth and production of crops. Finger millet is an important cereal grown in many arid and semi-arid areas of the world characterized by erratic rainfall and scarcity of good-quality water. Finger millet salinity stress is caused by the accumulation of soluble salts due to irrigation without a proper drainage system, coupled with the underlying rocks having a high salt content, which leads to the salinization of arable land. This problem is projected to be exacerbated by climate change. The use of new and efficient strategies that provide stable salinity tolerance across a wide range of environments can guarantee sustainable production of finger millet in the future. In this review, we analyze the strategies that have been used for salinity stress management in finger millet production and discuss potential future directions toward the development of salt-tolerant finger millet varieties. This review also describes how advanced biotechnological tools are being used to develop salt-tolerant plants. The biotechnological techniques discussed in this review are simple to implement, have design flexibility, low cost, and highly efficient. This information provides insights into enhancing finger millet salinity tolerance and improving production.

## Introduction

Finger millet [*Eleusine coracana* (L.) Gaertn.] is a valuable cereal crop that is a staple food crop by a large segment of population living in marginal areas of sub-Saharan Africa and Asia (Chivenge et al., 2015). Globally, in terms of cereal production in semi-arid regions, finger millet is ranked third after sorghum and pearl millet (Thilakarathna and Raizada, 2015). Its grain is rich in methionine, tryptophan, cysteine, tyrosine, calcium, phosphorous, and iron, making the crop an excellent nutritional source compared to other major cereals (Gupta et al., 2017). Finger millet has the potential to grow in marginal agroecological zones where other crops may not, and the grains have a long shelf life (Onyango, 2016). These attributes make it a valuable food crop and genetic resource that is critical for global food security. Its production is 4.5 million tons per annum with 2.0 million tons being produced in Africa (Sakamma et al., 2018). Given that new finger millet products such as bakery products, snacks, pasta, and sweet products are becoming increasingly popular, the demand for this crop is steadily increasing (Onyango, 2016). Furthermore, finger millet is a raw material for ethanol production (Tekaligne et al., 2015). The increasing global demand necessitates the development of advanced improvement techniques addressing complex attributes such as abiotic stresses that constrain its production.

Among the abiotic stresses constraining finger millet production is soil salinity, which is a devastating environmental stress factor that has a substantial negative impact on crop quality and production (Hema et al., 2014). In general, salinity causes osmotic stress which leads to physiological changes including membranes interruption, nutrient imbalance, damage to the ability to detoxify reactive oxygen species (ROS), decrease in photosynthetic activity, decrease in stomatal aperture, and changes in the antioxidant enzymes (Rahnama et al., 2010; Shahzad et al., 2021). The excess uptake and accumulation of Na^+^ and Cl^–^ ions into plant tissues result in severe ion imbalance and functional disorders. Elevated concentration of Na^+^ ions in the tissues impedes the uptake of elements essential for growth and development, such as K^+^ ions, thereby leading to reduced productivity and possible plant death (James et al., 2011; Shah et al., 2021).

Seed germination and seedling establishment stages in finger millet are highly affected by salinity stress (Hema et al., 2014). Many reports have shown that salinity stress substantially affects impacts seedling and root growth, levels of ion content and relative water content, photosynthetic pigments, proline content, levels of membrane peroxidation, and the amount of reducing sugars and total proteins (Kumar and Khare, 2016; Sarabi et al., 2017; Dugasa et al., 2020; Mukami et al., 2020). Although there is limited information available on yield loss of in large-scale producing areas, pot studies have reported genotypic variability in the grain yield loss among different multiple varieties (Krishnamurthy et al., 2014). The current review outlines the recent advanced strategies used in finger millet salinity stress management.

## Finger Millet Physiological and Biochemical Response to Salinity Stress

Plants respond to salinity stress by generating ROS via the processes of photosynthesis, photorespiration, and mitochondrial respiration (Sharma et al., 2013). ROS trigger oxidative damage to numerous cell components including membrane lipids, proteins, nucleic acids, and chlorophyll (Wang et al., 2012). Plants defend themselves from oxidative damage due to ROS through non-enzymatic and enzymatic defense mechanisms. Some of ROS-scavenging enzymes in plants include superoxide, ascorbate peroxidase, guaiacol peroxidase, dismutase, catalase, and glutathione reductase. These enzymes help in suppressing toxic ROS within cells (Misra and Gupta, 2005). Non-enzymatic defense mechanism includes osmotic adjustment, ion-selective absorption, and compartmentalization. Plants increase their osmotic potential by accumulating friendly organic solutes such as organic acids, carbohydrates, and quaternary ammonium compounds such as glycine betadine and proline (Ashraf and Foolad, 2007). Carbohydrates act as water replacement molecules (Crowe et al., 1987), minimize ROS-associated molecular alterations (Berjak et al., 2007), and ameliorate the concentration effects of salts and ions accumulated in the vacuole (Munns, 2002).

Earlier studies on the finger millet salinity tolerance primarily relied on the screening of salt-tolerant cultivars under saline conditions (Krishnamurthy et al., 2014; Rahman et al., 2014; Mukami et al., 2020). Various biochemical and physiological studies have reported that salt-tolerant cultivars, when exposed to saline conditions, are not adversely affected in terms of germination, shoot length, root length, biomass, Na^+^ and K^+^ ratio, total soluble sugars, membrane stability, and chlorophyll content (Rahman et al., 2014; Taïbi et al., 2016; Ishikawa and Shabala, 2019; Mukami et al., 2020). In their study, Mukami et al. (2020) reported a less lower reduction of germination rate in salt-tolerant than in salt-sensitive cultivars. Salt-tolerant cultivars also displayed a lower root/shoot growth retardation and had a slightly higher growth, low Na^+^ to K^+^ ratio in leaves and shoots, and higher amounts of total soluble sugars in leaves compared to salt-sensitive plants (Rahman et al., 2014). Several studies suggest that chlorophyll content is a biochemical marker of salt tolerance in plants (Taïbi et al., 2016; Ishikawa and Shabala, 2019). Salt-tolerant finger millet varieties have reported increased or unchanged chlorophyll levels under salinity conditions, whereas chlorophyll content decreased in salt-sensitive plants (Hema et al., 2014; Mahadik and Kumudini, 2020; Mukami et al., 2020). Plants use compatible solute accumulation to counter-attack the adverse effects of ROS. Proline is among the compatible solutes employed, and it acts as an osmoprotectant in plants under salinity stress as it preserves the integrity of the membrane and alleviates oxidative burst in plants (Rao et al., 2013; Rasool et al., 2013). In their study, Mahadik and Kumudini (2020) reported higher levels of proline accumulation in salt-tolerant cultivars than in salt-sensitive plants. Although determining the physiological and biochemical response of finger millet to salinity stress is vital for breeding and selecting salinity tolerant varieties, screening is time-consuming, and it is expensive for breeders to produce new salinity-tolerant varieties, because the biochemical and physiological traits obtained are highly variable owing to genetic heterogeneity response to salinity. Furthermore, the experiments are subject to environmental pressures and vulnerable to human error, resulting in unclear findings. Current research should strategically concentrate on new biotechnological strategies such as the use of genetic engineering, altering gene expression, and transcriptional control for the development of salt-tolerant finger millet cultivars.

## Finger Millet Molecular Response to Salinity Stress

In order to survive salinity stress, at the molecular level, plants activate a variety of genes and gene networks that encode numerous proteins that help them adjust and adapt to salinity stress. In one study, the salinity-sensitive leaf transcriptomes of multiple finger millet genotypes were sequenced. Salinity-sensitive genes were discovered after mapping and annotation of finger millet transcripts against rice gene models (Rahman et al., 2014). These include genes encoding the transporters, vacuolar ATP synthase, transcription factors, cell signaling molecules, osmoprotectant, biosynthesis of solutes that are compatible, and biosynthesis of phytohormones and carbohydrates (Table 1). Among the important transporter genes identified were the genes encoding ATP-binding transporters. Although the role of ATP-binding cassette transporters in plant salt tolerance is unknown, they have been confirmed to be involved in Arabidopsis salinity stress tolerance (Kim et al., 2010). In the presence of salt stress, ion homeostasis can be maintained by these transporters (Jiang et al., 2010). Other important findings of the study were the genes encoding the aquaporin proteins. Several studies have shown that plants use aquaporin proteins to help them cope with salt stress (Horie et al., 2011; Sun et al., 2017; Yepes-Molina et al., 2020). These proteins help preserve water balance under salinity stress by facilitating the flow of water through cellular membranes (plasma and vacuolar membranes). Signal amplification has a major impact on a plant’s ability to thrive in abiotic stress situations (Deokar and Tar’an, 2016). Genes encoding various signaling molecules including gibberellin 20-oxidase 2, serine/threonine kinase, calcium/calmodium-dependent protein kinase, receptor protein kinase, and receptor-like serine/threonine protein kinase were identified in the study (Table 1). The activation of these signaling elements influences the phosphorylation and dephosphorylation events controlling the stress signaling process, which could be associated with the higher level of salinity tolerance demonstrated by salt-tolerant finger millet genotypes. Stress-induced genes are involved not only in the defense of cells from stress through the development of essential metabolic proteins but also in gene regulation, such as transcription factors (TFs), which regulate the expression of multiple downstream target genes. Phytohormones play a vital role in curbing stress responses and adaptation, either by reducing or by mitigating the negative effects of salinity stress. Two important genes (Table 1) encoding brassinosteroid were identified in the study. Brassinosteroids are a class of phytohormones that promote growth in plants (Bishop, 2003) and are therefore implicated in plant responses against abiotic stresses.

**TABLE 1:** Salinity response genes identified in finger millet (Rahman et al., 2014).

**Transporters**	
LOC_ Os11g39020 LOC_Os02g32690 LOC_Os01g24010	ATP-binding cassette (ABC) transporters
**ATP synthase**	
LOC_ Os03g14690 LOC_Os10g10500 LOC_Os04g55040	Vacuolar ATP synthase
**Proteins**	
LOC_Os02g57720 LOC_Os03g05290 LOC_Os07g26630 LOC_Os07g26690)	Aquaporin proteins
**Signaling molecules**	
LOC_Os03g42130 LOC_Os06g04880 LOC_Os02g41580 LOC_Os03g46770 LOC_Os03g62180 LOC_Os07g0381 LOC_Os03g56270 LOC_Os07g35140 LOC_Os02g09740 LOC_Os01g26390	Gibberellin 20 oxidase 2 Serine threonine kinase Calcium/calmodulin dependent protein kinases RNA recognition Table motif containing protein Lectin protein kinase family protein Lectin-like receptor kinase 7 Receptor protein kinase Receptor-like serine-threonine protein kinase STRUBBELIGRECEPTOR FAMILY 7 precursor TKL_IRAK_DUF26-lh.1—DUF26 kinases
**Transcription factors (TFs)**	
LOC_Os03g60080 LOC_ Os08g36790 LOC_Os07g02060	NAC domain bZIP WRKY29
**Phytohormones**	
LOC_Os03g16980 LOC_Os07g47700	Brassinosteroid biosynthesis
**Osmoprotectants**	
LOC_Os11g26790 LOC_Os08g32870 LOC_Os01g62900	Dehydrins Betaine aldehyde dehydrogenase Δ1-pyrroline-5-carboxylate synthetase. Proline biosynthesis
LOC_Os03g20120, LOC_Os01g07530, LOC_Os07g10840, and LOC_Os03g59430 LOC_ Os06g46340 LOC_Os08g20660	Glycosyl transferases biosynthesis of raffinose Glycosyl hydrolase family 31. Hydrolysis of melibiose into galactose and glucose Sucrose phosphate synthase 1. Photosynthetic sucrose synthesis

Plants also tend to overpower antagonistic salinity-induced dehydration by accumulating metabolites or compatible solutes. Genes encoding compatible solutes, including dehydrins, glycine betaine, and proline were reported in the study. These metabolites play a role in osmo-protection. Several studies have reported that the accumulation of glycine betaine and proline (amino acid) plays an adaptive role in protecting sub-cellular structures and mediating osmotic adjustment during salt stress conditions (Ahmad et al., 2013; Kibria et al., 2017). Plants accumulate soluble carbohydrates such as sucrose as a response to salinity (Gupta and Huang, 2014). Among the various genes identified were genes encoding glycosyl transferases. This enzyme is active in the raffinose biosynthetic pathway and acts as an osmoprotectant and anti-oxidant, shielding the plant from oxidative stress (Nishizawa-Yokoi et al., 2008). While previous research has provided valuable morphological, biochemical, and molecular insights into the response of finger millet to salinity stress, the recent technological advancement of single-cell transcriptional analysis through high-throughput sequencing has the capacity to provide exciting new knowledge that would be difficult or impossible to obtain via traditional means.

Owing to its adaptation to a semi-arid tropical environment, finger millet has been classified as a salinity-tolerant crop. Attempts have been made to characterize the primary genes involved in salinity tolerance and to use them in future applications. Jayaprakash et al. (1998) looked at the stress-sensitive genes expressed in finger millet under salinity stress. They discovered that finger millet subjected to various osmotic stress treatments had higher levels of *LEA2* and *LEA3* (late embryogenesis abundant proteins) as well as better recovery development. Increased levels of *LEA2* and *LEA3* genes were discovered during the study of stress-sensitive genes. In another study, Rahman et al. (2016) found that overexpression of the *EcNAC67* transcription factor increased rice salinity tolerance. Recently, a novel finger millet endoplasmic reticulum-specific *bZIP TF* gene (*EcbZIP17*) was isolated and overexpressed in tobacco (Ramakrishna et al., 2018). In comparison to wild-type plants, tobacco plants overexpressing *EcbZIP17* showed resistance to saline stresses.

The inadequacy of mapped genes related to salinity tolerance in finger millet has substantially hindered studies of salinity tolerance genetics in finger millet as compared to other major cereals. Finger millet salinity tolerance remains poorly understood owing to this drawback. There exist only a few preliminary reports on the detection and validation of salt-tolerant candidate genes in finger millet. While several salt-tolerant genes have been singled out in finger millet (Rahman et al., 2014), they have yet to be validated in other plants, including model plants such as *Arabidopsis thaliana.* Overexpression of such genes in other plants will aid in understanding their role in salt stress responses and may be used to develop improved finger millet cultivars through breeding.

## Finger Millet Improvement for Salinity Tolerance

Owing to the detrimental effects of salinity stress finger millet production, effective salinity management actions are required to guarantee global food security especially in sub-Saharan Africa and Asia where this crop is predominantly cultivated (Chivenge et al., 2015). Several attempts have previously been made to grow high-yielding finger millet cultivars using various conventional methods including hybridization (Bisht and Mukai, 2001) and mutation breeding (Ambavane et al., 2015). These traditional methods have been reported to be unsuccessful in the production of salinity-tolerant finger millet cultivars. This is due to the difficulty and complicated nature of these methods, and the fact that salinity tolerance is a complex phenomenon involving various cellular pathways, genetic controls, and responses to environmental fluctuations (Manavalan et al., 2009).

Thus, approaches such as genomics, transcriptomics, proteomics, and metabolomics are required to understand salinity tolerance. Omics studies have been used to understand salinity tolerance in a variety of plants: soybeans (Liu et al., 2016; Kim et al., 2017; Wang Y. et al., 2018), rice (Das et al., 2015), chickenpea (Vadez et al., 2012), cowpea (Chankaew et al., 2014), and pea (Leonforte et al., 2013). Transgenic approaches have facilitated the development of salt-tolerant plants with higher yield and productivity. These approaches involve the identification and expression of candidate genes conferring salt tolerance. Several studies have reported various potential genes conferring salinity tolerance in plants: *UGT76E12* (Chen et al., 2019), *PtDRS1* (Mohammadi et al., 2018), *MdY3IP1* (Yu et al., 2018), cysteine protease (Zheng et al., 2018), *AtHDG11* (Banavath et al., 2018), *codA* (Baloda et al., 2017), *NHX1* and *bar* (Kumar et al., 2017), *rstB* (Zhang and Wang, 2015), and *GsZFP1* (Tang et al., 2013). Exogenous genes that are expressed to generate salt-tolerant finger millet have been identified in a few studies so far. Mahalakshmi et al. (2006) isolated a cDNA clone encoding a serine-rich protein from a cDNA library of salt-stressed *Porteresia coarctata* roots, dubbed *P. coarctata* serine-rich-protein (*PcSrp*) encoding gene. *PcSrp* expression was discovered in the salt-stressed roots and rhizome of *P. coarctata*. To determine its function, the *PcSrp* gene was cloned downstream of the rice actin-1 promoter and introduced into finger millet using the particle-inflow-gun method. Under 250-mM NaCl stress, transgenic plants expressing *PcSrp* were able to mature and set seed. The untransformed control plants, contrast, did not withstand similar salt stress. Transgenic plants’ stressed roots invariably accumulated higher Na^+^ and K^+^ ion contents than untransformed plants “roots, while transgenic plants” shoots accumulated lower amounts of both ions. Hema et al. (2014) expressed the *mtlD* (mannitol-1-phosphate dehydrogenase) gene in finger millet in another study. Salinity tolerance testing revealed that transgenic plants expressing the *mtlD* gene grew faster than wild-type plants under salinity stress. Similarly, Anjaneyulu et al. (2014) genetically engineered finger millet plants with the *SbVPPase* gene isolated from *Sorghum bicolor* and studied the biochemical and physiological parameters of control and transgenic plants. In control plants, the relative water content, plant height, leaf expansion, plant length, width, and grain weight were all severely reduced, and flowering was delayed by 20%. By contrast, transgenic plants had higher proline, chlorophyll content, enzyme activity, and lower malondialdehyde levels (MDA). These three examples demonstrate how genetic engineering can substantially aid salinity stress management in finger millet. As effective regeneration protocols in finger millet have been developed (Mukami et al., 2018; Ngetich et al., 2018) and a wide range of salinity-tolerance genes have been reported (Rahman et al., 2014; Mohammadi et al., 2018; Yu et al., 2018; Zheng et al., 2018; Chen et al., 2019), more research should be undertaken to exploit the available genes for improving this crop against salinity stress.

## Salinity Management Using Secondary Metabolites and Microorganisms in Finger Millet

Cellular water homeostasis and ionic balance are essential for the optimal functioning of physiological, biochemical, and molecular processes in plants. Soil salination disrupts water uptake, triggering plant cell ionic imbalances (accumulation of Na^+^ and Cl^–^), osmotic stress, and oxidative damage, which in turn impedes the growth and development of the plants (Hossain and Dietz, 2016). To acclimatize and adapt to the saline stress conditions, plants respond with complex and elaborate strategies to address ion homeostasis, osmolyte biosynthesis, toxic ions compartmentation, and ROS-scavenging systems, which results in accumulation or decline of specific secondary metabolites. Exposure of different rice genotypes to different severity of salt stress proportionately increased the terpene emissions in a time-dependent manner. The salt-sensitive genotype emitted higher volumes of terpenes than the tolerant genotype (Chatterjee et al., 2018). Similarly, phenolic compounds are reported to increase in response to salt stress *in Thymus vulgaris* L. and *Thymus daenensis* Celak (Bistgani et al., 2019). Anthocyanins, which act as modulators of excitation pressure, have been shown to be key regulators of salt-stressed plant species such as wheat (Mbarki et al., 2018) and *Brassica napus* (Kim et al., 2017). In contrast, salt-sensitive species, such as tomato, demonstrate decreased anthocyanin levels (Daneshmand et al., 2010). Proline accumulation in plants is related to water deficit and salinity stress, where it acts as an osmolyte for osmotic adjustment, stabilizes membranes and proteins, scavenges free radicals, and buffers cellular redox potential under stress conditions (Boscaiu Neagu et al., 2012). An increased level of endogenous proline accumulation in plants is correlated with enhanced salt tolerance (Sripinyowanich et al., 2013), and has been reported in a number of plant species such as *Pisum sativum* (Ozturk et al., 2012), *Glycine max* (Weisany et al., 2012), *Cucumis melo* (Sarabi et al., 2017), *Oryza sativa* (Kibria et al., 2017), and *Eleusine coracana* (Mahadik and Kumudini, 2020; Mukami et al., 2020).

Glycine betaine, which is widely distributed in microorganisms, higher plants, and animals, is one of the most common betaines found in plants and is involved in osmotic adjustment (Al Hassan et al., 2016). Glycine betaine has been reported to act as an osmoprotectant by reducing ROS and hence stabilizing cellular macromolecules under adverse conditions (Ahmad et al., 2013). Many studies have revealed that salt-tolerant genotypes/species usually accumulate more glycine betaine than sensitive ones when subjected to salt stress. Such increased accumulation of glycine betaine has been reported to correlate with salt tolerance in many plants such as barley (Chen et al., 2007) and rice (Cha-um et al., 2007). When the *SoBADH* gene from *Spinacia oleracea* was transferred into the sweet potato cultivar, the transgenic lines exhibited improved tolerance to salt stress (Fan et al., 2012). Under normal and stressful conditions, the chloroplastic *BADH* activity and glycine betaine accumulation were elevated, resulting in the maintenance of cell membrane integrity as well as increased photosynthetic activity and antioxidant enzyme activities. Similarly, transgenic tobacco plants expressing the *OsBADH1* gene accumulated glycine betaine, resulting in normal seed germination and morphology, as well as normal transgenic line growth rates under salt stress (Hasthanasombut et al., 2010). Based on these findings, it is obvious that increasing glycine betaine production is a viable and effective way to improve salt stress tolerance. Other than amino acids, salt-stressed plants have been reported to accumulate carbohydrates such as sugar and starch. When rice seedlings were exposed to NaCl, the sugar content of the shoots increased considerably, whereas the starch content of the root seedlings decreased. In roots, the total, reducing, and non-reducing sugar content increased (Amirjani, 2011). In a recent study, the impact of salinity treatment triggered substantial elevation in reducing sugar amounts in stressed finger millet plants when compared to the control experiments (Mukami et al., 2020). The accumulated carbohydrates perform key functions in stress mitigation, including osmoprotection, carbon storage, and ROS scavenging (Gupta and Huang, 2014).

For decades, soil microorganisms have been used in crop production (Hayat et al., 2010). They are primarily involved in the supply of nutrients to crops, the stimulation of plant growth through the processing of plant hormones, the regulation or inhibition of plant pathogen activities, the improvement of soil structures, and inorganic bioaccumulation or microbial leaching (Ramadoss et al., 2013). Plant growth-promoting rhizobacteria have been used in the cultivation of a number of plant species to improve crop quality by reducing the negative effects of salt stress on plant growth. In general, bacterial inoculation increases root and shoot length, biomass, and biochemical levels of chlorophyll, carotenoids, and protein (Tiwari et al., 2011). Several studies have demonstrated that applying plant growth rhizobacteria (PGPR) to plants improves their salt tolerance (Casanovas et al., 2002; Mayak et al., 2004; Barassi et al., 2006; Yao et al., 2010; Egamberdieva, 2012; Nia et al., 2012; Upadhyay et al., 2012; Ahmed et al., 2013; Ramadoss et al., 2013; Wang W. et al., 2018; Rafiq et al., 2020). To date, only one report is available on the use of PGPR in enhancing salinity tolerance in finger millet. Mahadik and Kumudini (2020) investigated the effects of fluorescent *Pseudomonas* strains (SPF-5, SPF-33, and SPF-37) isolated from saline regions on salinity sensitive finger millet seeds. In their study, finger millet seeds were treated with isolates and exposed to various concentrations (0–350 mM NaCl) of salt stress. Isolates were screened for growth-promoting characteristic qualities and growth parameters under greenhouse conditions. Under increased salt conditions, strain SPF-33 showed increased enzymatic antioxidant activity and increased proline content, lower lipid peroxidation and hydrogen peroxide, and increased plant height and spikelet number. Under 350-mM NaCl, treatment with SPF-37 increased germination, vigor index, plant height and the number of spikelets, total chlorophyll, phenolics, flavonoids, proteins, and relative water content of the leaf, considerably more than the control. These results demonstrated that fluorescent *Pseudomonas* strains SPF-33 and SPF-37 are potential PGPR for improving finger millet salinity tolerance. Currently, there is limited information on an array of rhizobacteria that can be used to mitigate salinity stress in finger millet as well as information on complex plant–microbial interactions in various agro-ecosystems. This knowledge is needed in order to implement this management strategy in finger millet. More work needs to be done to verify the current outcomes of PGPR usage and to improve our knowledge and understanding about the value of microbial agents. Furthermore, PGPR should be used in conjunction with other techniques to protect finger millet from salinity stress over time.

These examples clearly affirm the importance of plant secondary metabolites and microorganisms in mitigating salt stress in plants. Traditionally, managing salinity stress in agriculture has heavily relied on development of salt-tolerant crop varieties, a time-consuming, expensive, and difficult process for many crops. Bioeffectors (biostimulants), compounds of biological origin that are applied to embellish nutrient uptake, stimulate growth and enhance stress tolerance or quality traits of crops (Van Oosten et al., 2017), through stimulation of the plant’s metabolic and defense mechanisms. Exogenous application of several bioeffectors such as copper chlorophyllin (Cu-chl), a semi-synthetic water-soluble chlorophyll derivative (Islam et al., 2021), *Moringa oleifera* leaf extract (Desoky et al., 2018), and *Ascophyllum nodosum* extracts (Dell’Aversana et al., 2021) have been shown to enhance salinity stress tolerance in *A. thaliana*, sorghum, and tomato, respectively. Plant-based bioeffectors therefore form part of the sustainable approach to enhance salt stress tolerance in crops and could potentially address breeding handicaps, as they are versatile and are easy to apply in the field. In order to take full advantage of bioeffectors in compacting salinity stress, finger millet specificities and application techniques should be identified for optimum impact on stress protection. Equally, comprehensive and deeper knowledge of the functional mechanism of bioeffectors is important for their application in omics, system biology, and synthetic biology.

## Gene Pyramiding and Multiple Character Breeding to Enhance Salt Stress Tolerance

The advancement of plant genomics has played a key role in the release of vital genomic data for trait improvement in crops. Using a genomics approach, various genes that are involved in salinity stress have been identified and characterized in plant systems. For example, Chakraborty et al. (2012) studied differential gene expression in *Brassica* spp. and discovered a more efficient salt overly sensitive pathway made up of *SOS1, SOS2, SOS3*, and the vacuolar Na^+^/H^+^ antiporter. The conserved structure of these genes, as well as their intra and intergenic relatedness, was revealed by sequence analyses of partial cDNAs. Based on their findings, they concluded that the existence of an efficient *SOS* pathway, which results in a higher K/Na ratio, could be a major factor, among others, in deciding the salinity stress tolerance of *Brassica juncea* genotypes CS 52 and CS 54 as compared to Varuna and T 9. In another study, Zou et al. (2012) used transgenic rice plants overexpressing *OsHsp17.0* and *OsHsp23.7* to test stress tolerance. When exposed to mannitol and NaCl, both *OsHsp17.0-OE* and *OsHsp23.7-OE* transgenic lines showed higher germination potential than wild-type plants. Transgenic rice lines showed higher resistance to drought and salt stress than wild-type plants, according to a phenotypic study. Furthermore, under drought and salt stress conditions, transgenic rice plants had lower MDA and higher free-proline levels than wild-type rice lines. These findings showed that *OsHsp17.0* and *OsHsp23.7* are important in rice salt adaptation to salinity and dehydration stresses, and that they can be used to engineer rice that is drought and salt-tolerant.

It is now widely known that many plant traits are superintended by multigenes, especially pathways that result in biosynthesis of metabolites due to the intricate metabolic pathways involved in their biosynthesis, accumulation, metabolism, and catabolism (Ashraf et al., 2018). While single-gene transgenic technology has been widely used to improve plant salt tolerance, engineering using a single gene is often inadequate to trigger threshold expression of the metabolic products required. Given the complexity of some biosynthetic pathways and multiple traits involved in salinity stress, the efficacy of salt-tolerant genes can be improved by combining their presence in the same plant during crop improvement (Biradar et al., 2018). To achieve this goal, several multigene pyramiding approaches such as co-transformation of multiple genes and transgenic pyramiding by conventional hybridization have been developed to introduce multiple genes or complex metabolic pathways into plants. Although the use of conventional approaches such as sexual crossing is simple, it is labor-intensive and time−consuming and the method is unsuitable for sexually incompatible plants. A novel strategy known as speed breeding (discussed below) has been developed to accelerate the plant breeding period. Although delivery of multiple genes via *Agrobacterium*-mediated transformation has been achieved, it becomes a challenge with an increase in the number of transgenes and the size of the transfer-DNA (T-DNA), and has the potential of gene silencing if the same promoter is repetitively used. A biolistic transformation system can simultaneously introduce many transgenes encoding for multiple strains. However, this system comes with the limitations of a complex genome integration of transgenes and an unstable linkage inheritance between transgenic generations (Chen et al., 1998; Liu et al., 2018).

Several examples have demonstrated that co-expression of genes in the same plant can improve salt tolerance in plants: rice (Gupta et al., 2018), *Spartina alterniflora* (Biradar et al., 2018), potato (Shafi et al., 2017), Arabidopsis (Pehlivan et al., 2016), sweet potato (Yan et al., 2016), *Festuca arundinacea* (Ma et al., 2014), tomato (Viveros et al., 2013), and tobacco (Singla-Pareek et al., 2003). Only one study has reported that the co-expression of genes increases salinity tolerance in finger millet. Using *Agrobacterium*-mediated transformation, Jayasudha et al. (2014) co-expressed *PgNHX1* from *Pennisetum glaucum* and *AVP1* from *A. thaliana.* Compared to wild-type plants, dual-transgenic plants displayed higher salt tolerance to salt stress. Although this is a single study, and in cognizance of previous reports, their findings affirm that gene stacking is the most effective technique for providing plants with long-term salt-stress tolerance. In the literature, most plant-breeding approaches for abiotic stress resistance are hinged on single gene introgression into a recipient genome whose developed defense starts to decline after a short period of time owing to complex interactive effects brought about by the changing climate. As salinity stress is a polygenic trait, the stability of salinity tolerant cultivars can be lost when tolerance is based on one major gene. Based on this observation, pyramiding multiple genes which confers resistance against several stresses into a single plant should now be accentuated. A transgenic strategy for developing finger millet with sustainable and stable tolerance should involve the screening of several potential salinity-tolerance genes and the pyramiding of the desirable ones. The use of genetic engineering techniques to combine multiple genes into a single finger millet cultivar is a promising approach for developing tenacious and superior tolerance, particularly when candidate genes come from diverse gene clusters and should be emphasized. Although gene pyramiding appears to be a promising technique for alleviating salt stress in plants, potential challenges include reduced effectiveness of stacking genes, individual gene mutations, gene silencing, genotype–environmental interactions, a combination of various sources of genetic material with non-redundant mode of action, and the lengthy time taken to produce a successful variety, especially by seed companies. New methods, such as genome editing, have helped to overcome these obstacles.

## CRISPR/Cas Genome Editing and Precision Breeding for Salinity Stress Tolerance in Finger Millet

The novel clustered regularly interspaced short palindromic repeats (CRISPR)-CRISPR-associated protein (Cas) system has emerged as an effective robust tool for site-specific genome editing and precise induction of mutagenesis in many organisms including plants. Given its precision, efficiency, simplicity, and high cost-effectiveness, the CRISPR/Cas system has been extensively applied to the edit genomes of several crops including those closely related to finger millet such as foxtail millet (Cheng et al., 2021; Zhang et al., 2021), sorghum (Liu et al., 2019; Char et al., 2020), rice (Tang et al., 2017; Wang L. et al., 2017; Wang M. et al., 2017; Dong et al., 2020), and wheat (Liang et al., 2017; Sánchez-León et al., 2018), to develop crop varieties with enhanced resistance to biotic and abiotic stresses as well as other traits. On specific application of CRISPR/Cas technology toward development of crops with enhanced tolerance to salinity stress, Zhang et al. (2019) reported improvement of the rice salinity tolerance by engineering a Cas9-OsRR22-gRNA expressing vector, targeting the OsRR22 gene in rice. In their results, homozygous mutant lines displayed substantially higher salinity tolerance than wild-type plants. More recently, Bouzroud et al. (2020) focused on the involvement of SlARF4 gene in tomato salinity tolerance and osmotic stress. A CRISPR/Cas9-induced SlARF4 mutant showed similar growth and stomatal responses to ARF4-as plant lines previously generated and well-characterized by Sagar et al. (2013). These examples demonstrate the potential applicability of CRISPR/Cas technology in finger millet. To the best of our knowledge, no literature exists on the application of CRISPR/Cas technology to finger millet. Since the first report on its application in plants in 2013 (Feng et al., 2013; Li et al., 2013; Nekrasov et al., 2013; Shan et al., 2013), his technology has revolutionized crop breeding, enabling plant breeders to precisely control the specific introduction of targeted mutagenesis.

The fundamental principle of CRISPR/Cas and other genome editing tools, such as meganucleases (Puchta et al., 1993), zinc-finger nucleases (Wright et al., 2005), and transcription activator-like effector nucleases (Christian et al., 2010), is to employ a sequence-specific nuclease protein to induce a DNA double-strand break (DSB) at a target-specific locus of the genome. After cleavage, either the error-free homologous directed repair (HDR) pathway or the non-homologous end joining (NHEJ) mechanism (which is error-prone) repairs the DSB, introducing genetic mutations. NHEJ repair predominantly occurs during the G1 phase although the mechanism is also postulated to occur throughout the cell cycle. The HDR process is most dominant during S and G2 phases of cell division. Repair of DSB in somatic plant cells favors NHEJ more than the HDR (Schmidt et al., 2019). All the aforementioned genome editing platforms have shown an impressive capacity for plant genome editing. However, with the exception of the CRISPR/Cas system, all the aforementioned tool platforms require complex protein engineering, which is costly, time-consuming, and of doubtful precision, limiting their applicability. The principal CRISPR/Cas system, which is adopted from a bacterial immune system response against invading viruses, consists of CRISPR repeat-spacers and Cas proteins, which is an RNA-mediated adaptive immune system against viruses and other invasive non-host genetic elements by cleaving the invader’s foreign nucleic acid material. Currently, CRISPR/Cas systems are divided into two major classes, which have been further classified into six subdivisions as per the properties of their respective Cas genes (Chen et al., 2019). Editing of organisms’ genomes by the CRISPR system was therefore formulated based on DNA interference by guided RNA (gRNA). Given its ability to induce precise nucleotide mutations, and its global acceptance by plant biotechnologies, CRISPR/Cas technology has the potential to have a substantial positive impact on agriculture including development of crop cultivars resistant to salinity stress.

Theoretically, the CRISPR/Cas system can be used to manipulate all genomes with high accuracy. However, the gRNA can find complementary positions within the genome and cause off-targets, although they are rare in plants (Peterson et al., 2016). To avoid unexpected mutations, care should be taken when choosing the Cas protein and the design of the gRNA. The designing of gRNA requires a reference genome of the crop of interest. Unfortunately, a complete assembled genome of finger millet has not yet been released. This has considerably hampered the application of the CRISPR/Cas genome editing tool in finger millet. Although whole-genome draft sequencing and assembly of finger millet was released several years ago (Hittalmani et al., 2017; Hatakeyama et al., 2018), validation of sequence reads data and annotation of key genes is yet to be concluded, leaving only raw reads in the database. Furthermore, the genome has not been uploaded in major gRNA designing tools and therefore the designing of gRNAs is limited. More work is therefore urgently needed to invigorate the use of the crop’s genomics applications. Other impediments in the application of the CRISPR/Cas system in finger millet are a lack of efficient *in vitro* regeneration and transformation protocols that are cultivar-independent. Although attempts have been made for tissue culture regeneration and transformation of finger millet using different explants and different delivery methods, their efficiency is cultivar-dependent with low regeneration and transformation efficiency (Kothari et al., 2004; Ignacimuthu and Ceasar, 2012; Satish et al., 2017; Ngetich et al., 2018). Currently, there is no report available for *in vitro* regeneration through another culture, protoplast culture, and protoplast fusion in finger millet.

## Potential Improvement of Finger Millet Salt Stress Resistance Using Synthetic Biology

Agriculture and applications of bioengineering techniques must be used to help feed the burgeoning global population in a sustainable manner under climate change perturbations (Wurtzel et al., 2019). The most recent technique is synthetic biology, which, if well adopted like the CRIPSR/Cas genome editing system, can play a crucial role in mitigating complex abiotic challenges in crop cultivation, including crop salinity stress. Although synthetic biology can be classified as an offshoot of genetic engineering, it completely differs from the “cutting-and-pasting” of genetic material from one organism into another. It involves designing a complete organism from scratch using computational and mathematical modeling and quantitative functional characterization for useful purposes (Preston, 2013; Wurtzel et al., 2019; Roell and Zurbriggen, 2020).

Over the years, plants have evolved elaborate and complex mechanisms for avoiding or tolerating the effects of salinity stress. Avoidance mechanisms prevent exposure and include the capability to reduce sodium ions accumulation in the cell through water-loss minimization and optimization of water uptake. Salinity tolerance allows plants to endure elevated cellular sodium ion concentrations by preserving cell turgor and raising protoplasmic resistance (Soliman et al., 2018). With an increasing number of genomics resources available for various plant lineages displaying different approaches and variation in salinity-stress avoidance or tolerance (Bhardwaj et al., 2014; Razali et al., 2018; Chanwala et al., 2020; Noori et al., 2021), systems biology, a technique that utilizes genome-scale analyses of molecules and their interplay (Shah et al., 2017), is emerging as an attractive approach to connect genes to salinity-stress avoidance or salinity-tolerance traits. Therefore, the utilization of synthetic biology technologies in developing finger millet plant lines impervious to salinity stress represents not only the accumulative insertions of transgenes but also the directed construction of entirely new metabolic pathways and networks, physiological traits, and growth and developmental control strategies. At present, the application of synthetic biology approaches in plants has not grown in tandem with bacterial and mammalian systems, where these tools are already redesigning fundamental research in many aspects (Roell and Zurbriggen, 2020). Despite its high potential, most current developments have not been translated to “outside-the-lab bench” application spaces, which are completely diverse and variable compared to the controlled laboratory settings. Another challenge in the application of synthetic biology tools in agriculture is the time and cost involved in *in vitro* propagation, genetic manipulation, and screening of crops. While there has been a boost to plant biotechnology following the development of novel and revolutionary techniques such as CRISPR/Cas-mediated genome editing and speed breeding, the whole sequencing and annotation of orphaned crops like finger millet and the application and growth of synthetic biology as a field remain a challenge. Nevertheless, the adoption of synthetic biology tools will be vital in remodeling future progress in agricultural biotechnology especially when dealing with multigenic stresses such as salinity.

## Speed Breeding Technique and Potential Use for Salinity Management in Finger Millet

The technology advancement in the last three decades has exposed contemporary plant breeders to a plethora of innovative tools, such as genomic selection (Werner et al., 2020), high-throughput phenotyping (Hu et al., 2020), genome editing (Miladinovic et al., 2021), enviromics (Resende et al., 2021), and speed breeding (Watson et al., 2018), for application and integration into crop improvement pipelines in the face of the adverse environmental conditions caused by climate change and the consequently increased occurrence of biotic and abiotic stresses. Speed breeding methods that shorten plant generation times have been regarded as a powerful and revolutionary tool for accelerating crop research and breeding. The principle of speed breeding is the use of optimum temperature, light intensity, and photoperiod to hasten growth and development (Ghosh et al., 2018; Watson et al., 2018). Combining a large number of polygenic traits using conventional breeding methods is a considerable challenge (Breseghello and Coelho, 2013). From this perspective, speed breeding provides a precise breeding tool for improving specific traits in plants during the breeding cycle. Since their unveiling, species-specific speed-breeding protocols have been used to achieve up to six generation cycles in a year for spring wheat, durum wheat, barley, chickpea, and pea, as well as four generations for canola, as opposed two to three under glasshouse conditions (Watson et al., 2018), demonstrating an effective tool for reducing breeding time. However, a speed-breeding protocol for finger millet has yet to be developed. Delivering speed breeding in finger millet requires optimization in a simplified and affordable manner. Attributes like salinity tolerance in crops could be enhanced by selecting the outstanding hybrid progeny harboring desired traits through back-crossing (Dolferus et al., 2011). To curtail undesirable phenotype combinations, the desired trait can also be introduced into a recipient plant line by backcrossing selected progeny with recipient lines for several generations (Caligari and Forster, 2015). Rana et al. (2019) developed a new rice cultivar that is highly tolerant to salt stress, YNU31-2-4, through efficient marker-assisted selection coupled with speed breeding. Results from their study demonstrate that breeding system in combination with other breeding techniques can be utilized as an effective and rapid way to mitigate salinity stress in crops including finger millet, which can potentially boost global food production to meet the growing population’s food security needs.

## Agronomic Management of Salinity Stress

Agronomic methods to reduce salinity stress during cultivation can be directed on either soil or crops. The simplest approach to lower soil sanity is to increase the soil water content, which can be achieved through frequent irrigation especially the modern irrigation systems with high efficiency. Increasing of the soil water content reduces the salt concentration to level which crops can withstand. However, this approach is not feasible for smallholder finger millet farmers. Further, finger millet is grown in arid and semi-arid regions of the world. Dry periods of finger millet cultivation are the most critical period for salinity stress. Survival of crops during such periods can be increased by the application of calcium nitrate or chloride because Cl^–^ ions move the Na^+^ ions from soil colloids which leached by rains or irrigation (Mariani and Ferrante, 2017). Similar effects are also reached by application magnesium. Therefore, the application of fertilizers containing high calcium and magnesium saline soils improves the soil structure and provides a suitable environment for roots and plant growth. At the crop physiology level, cellular Cl^+^ ions inhibit the membranes’ sodium channel and decrease cytosolic salt accumulation rate, thereby allaying plant salinity stress. Moreover, if the calcium used is in the form of nitrates, the Cl^–^ ions compete with Na^+^ ions for accumulation in the cytosol vacuoles. The application of nitrates to sodic soils may reduce salt uptake, although this aspect requires validation.

## Conclusion and Future Perspectives

Many studies have emphasized the necessity for crop production to double by 2050 to meet the anticipated demands of a burgeoning global population. At present, soil salinity is the most serious threats among abiotic stresses caused by climate change and agronomic practices that impede crop production. Although meaningful gains have been made in breeding salt-tolerant crops, more work is needed. Salinity tolerance is a polygenic trait, and this is further complicated in several types of abiotic stress, such as drought, high temperatures, and nutrient deficiencies and toxicities, that may simultaneously impact the crop, making breeding for salt-tolerant cultivars a challenge. From this review, it can be summarized that finger millet production is adversely affected by salt stress, which alters its physiological, biochemical, and molecular mechanisms. The genes, metabolites, and pathways responsible for the diverse mechanisms of salinity tolerance in finger millet must be profiled, taking advantage of the discovery of the whole genome sequence of finger millet. Based on the advancement of biotechnological tools at present, multidisciplinary approaches are encouraged for the development of salt-tolerant finger millet cultivars. Several new techniques such as genomic selection, high-throughput phenotyping, genome editing using the CRISPR/Cas, speed breeding, and synthetic biology have recently attracted attention among scientists and plant breeders globally. All these strategies promise to revolutionize comprehensive trait prediction and integration of various salinity management options, including agronomic approaches, conventional breeding, and modern biotechnological advances, for the sustainable improvement of finger millet yield and nutritional quality under salt stress.

## Author Contributions

WM conceptualized and critically edited the manuscript for publication. WM and AM undertook the literature review and analysis and wrote the manuscript. Both authors contributed to the article and approved the submitted version.

## Author Disclaimer

Views expressed herein do not necessarily reflect the official opinion of the funding organization.

## Conflict of Interest

The authors declare that the research was conducted in the absence of any commercial or financial relationships that could be construed as a potential conflict of interest.

## Publisher’s Note

All claims expressed in this article are solely those of the authors and do not necessarily represent those of their affiliated organizations, or those of the publisher, the editors and the reviewers. Any product that may be evaluated in this article, or claim that may be made by its manufacturer, is not guaranteed or endorsed by the publisher.

## References

[B1] Ahmad M. Zahir Z. A. Khalid M. Nazli F. Arshad M. (2013). Efficacy of Rhizobium and *Pseudomonas* strains to improve physiology, ionic balance and quality of mung bean under salt-affected conditions on farmer’s fields. *Plant Physiol. Biochem.* 63 170–176. 10.1016/j.plaphy.2012.11.024 23262185

[B2] Ahmed F. Baloch D. M. Hassan M. J. Ahmed N. (2013). Role of plant growth regulators in improving oil quantity and quality of sunflower hybrids in drought stress. *Biologia* 59 315–322.

[B3] Al Hassan M. Morosan M. López-Gresa M. D. P. Prohens J. Vicente O. Boscaiu M. (2016). Salinity-induced variation in biochemical markers provides insight into the mechanisms of salt tolerance in common (*Phaseolus vulgaris*) and runner (*P. coccineus*) beans. *Int. J. Mol. Sci.* 17:1582. 10.3390/ijms17091582 27657045 PMC5037847

[B4] Ambavane A. R. Sawardekar S. V. Sawantdesai S. A. Gokhale N. B. (2015). Studies on mutagenic effectiveness and efficiency of gamma rays and its effect on quantitative traits in finger millet (*Eleusine coracana* L. Gaertn). *J. Radiation Res. Appl. Sci.* 8 120–125. 10.1016/j.jrras.2014.12.004

[B5] Amirjani M. R. (2011). Effect of salinity stress on growth, sugar content, pigments and enzyme activity of rice. *Int. J. Botany* 7 73–81. 10.3923/ijb.2011.73.81

[B6] Anjaneyulu E. Reddy P. S. Sunita M. S. Kishor P. B. K. Meriga B. (2014). Salt tolerance and activity of antioxidative enzymes of transgenic finger millet overexpressing a vacuolar H+-pyrophosphatase gene (SbVPPase) from Sorghum bicolor. *J. Plant Physiol.* 171 789–798. 10.1016/j.jplph.2014.02.001 24877670

[B7] Ashraf M. A. Iqbal M. Rasheed R. Hussain I. Riaz M. Arif M. S. (2018). Environmental stress and secondary metabolites in plants: an overview. *Plant Metab. Regul. Under Environ. Stress* 2018 153–167. 10.1016/B978-0-12-812689-9.00008-X

[B8] Ashraf M. F. M. R. Foolad M. (2007). Roles of glycine betaine and proline in improving plant abiotic stress resistance. *Environ. Exp. Bot.* 59 206–216. 10.1016/j.envexpbot.2005.12.006

[B9] Baloda A. Seema Madanpotra S. Jaiwa P. K. (2017). Transformation of mungbean plants for salt and drought tolerance by introducing a gene for an osmoprotectant glycine betaine. *J. Plant Stress Physiol.* 3:5. 10.19071/jpsp.2017.v3.3148

[B10] Banavath J. N. Chakradhar T. Pandit V. Konduru S. Guduru K. K. Akila C. S. (2018). Stress inducible overexpression of AtHDG11 leads to improved drought and salt stress tolerance in peanut (*Arachis hypogaea* L.). *Front. Chem.* 6:34. 10.3389/fchem.2018.00034 29552555 PMC5840212

[B11] Barassi C. A. Ayrault G. Creus C. M. Sueldo R. J. Sobrero M. T. (2006). Seed inoculation with *Azospirillum mitigates* NaCl effects on lettuce. *Sci. Horticulturae* 109 8–14. 10.1016/j.scienta.2006.02.025

[B12] Berjak P. Farrant J. M. Pammenter N. W. (2007). “Seed desiccation-tolerance mechanisms,” in *Plant Desiccation Tolerance*, eds Jenks M. A. Wood A. J. (Wallingford: CAB International Press).

[B13] Bhardwaj A. R. Joshi G. Pandey R. Kukreja B. Goel S. Jagannath A. (2014). A genome-wide perspective of miRNAome in response to high temperature, salinity and drought stresses in *Brassica juncea* (Czern) L. *PLoS One* 9:e92456. 10.1371/journal.pone.0092456 24671003 PMC3966790

[B14] Biradar H. Karan R. Subudhi P. K. (2018). Transgene pyramiding of salt responsive protein 3-1 (SaSRP3-1) and SaVHAc1 from *Spartina alterniflora* L. enhances salt tolerance in rice. *Front. Plant Sci.* 9:1304. 10.3389/fpls.2018.01304 30258451 PMC6143679

[B15] Bishop G. J. (2003). Brassinosteroid mutants of crops. *J. Plant Growth Regulation* 22 325–335. 10.1007/s00344-003-0064-1 14676972

[B16] Bisht M. S. Mukai Y. (2001). Genomic in situ hybridization identifies genome donor of finger millet (*Eleusine coracana*). *Theoret. Appl. Genet.* 102 825–832. 10.1007/s001220000497

[B17] Bistgani Z. E. Hashemi M. DaCosta M. Craker L. Maggi F. Morshedloo M. R. (2019). Effect of salinity stress on the physiological characteristics, phenolic compounds and antioxidant activity of *Thymus vulgaris* L. and *Thymus daenensis* Celak. *Industrial Crops Products* 135 311–320. 10.1016/j.indcrop.2019.04.055

[B18] Boscaiu Neagu M. T. Donat Torres M. D. P. Llinares Palacios J. V. Vicente Meana Ó (2012). Stress-tolerant wild plants: a source of knowledge and biotechnological tools for the genetic improvement of stress tolerance in crop plants. *Notulae Botanicae Horti Agrobotanici* 40 323–327. 10.15835/nbha4028199

[B19] Bouzroud S. Gasparini K. Hu G. Barbosa M. A. M. Rosa B. L. Fahr M. (2020). Down regulation and loss of auxin response factor 4 function using CRISPR/Cas9 alters plant growth, stomatal function and improves tomato tolerance to salinity and osmotic stress. *Genes* 11:272. 10.3390/genes11030272 32138192 PMC7140898

[B20] Breseghello F. Coelho A. S. G. (2013). Traditional and modern plant breeding methods with examples in rice (*Oryza sativa* L.). *J. Agricultural Food Chem.* 61 8277–8286. 10.1021/jf305531j 23551250

[B21] Caligari P. D. Forster B. P. (2015). Plant breeding and crop improvement. *eLS*. 10.1002/9780470015902.a0002024.pub3

[B22] Casanovas E. M. Barassi C. A. Sueldo R. J. (2002). Azospirillum inoculation mitigates water stress effects in maize seedlings. *Cereal Res. Commun.* 30 343–350. 10.1007/BF03543428

[B23] Chakraborty K. Sairam R. K. Bhattacharya R. C. (2012). Differential expression of salt overly sensitive pathway genes determines salinity stress tolerance in Brassica genotypes. *Plant Physiol. Biochem.* 51 90–101. 10.1016/j.plaphy.2011.10.001 22153244

[B24] Chankaew S. Isemura T. Naito K. Ogiso-Tanaka E. Tomooka N. Somta P. (2014). QTL mapping for salt tolerance and domestication-related traits in *Vigna marina* subsp. oblonga, a halophytic species. *Theoretical Appl. Genet.* 127 691–702. 10.1007/s00122-013-2251-1 24370961

[B25] Chanwala J. Satpati S. Dixit A. Parida A. Giri M. K. anf Dey N. (2020). Genome-wide identification and expression analysis of WRKY transcription factors in pearl millet (*Pennisetum glaucum*) under dehydration and salinity stress. *BMC Genomics* 21:1–16. 10.1186/s12864-020-6622-0 32171257 PMC7071642

[B26] Char S. N. Wei J. Mu Q. Li X. Zhang Z. J. Yu J. (2020). An Agrobacterium-delivered CRISPR/Cas9 system for targeted mutagenesis in sorghum. *Plant Biotechnol. J.* 18:319. 10.1111/pbi.13229 31374142 PMC6953201

[B27] Chatterjee P. Kanagendran A. Samaddar S. Pazouki L. Sa T. M. Niinemets Ü (2018). Inoculation of *Brevibacterium* linens RS16 in *Oryza sativa* genotypes enhanced salinity resistance: impacts on photosynthetic traits and foliar volatile emissions. *Sci. Total Environ.* 645 721–732. 10.1016/j.scitotenv.2018.07.187 30031330 PMC6354898

[B28] Cha-um S. Supaibulwatana K. Kirdmanee C. (2007). Glycinebetaine accumulation, physiological characterizations and growth efficiency in salt-tolerant and salt-sensitive lines of indica rice *(Oryza sativa* L. ssp. indica) in response to salt stress. *J. Agronomy Crop Sci.* 193 157–166. 10.1111/j.1439-037X.2007.00251.x

[B29] Chen L. Marmey P. Taylor N. J. Brizard J. P. Espinoza C. D’Cruz P. (1998). Expression and inheritance of multiple transgenes in rice plants. *Nat. Biotechnol.* 16 1060–1064. 10.1038/3511 9831036

[B30] Chen L. Wang W. S. Liu Q. Dong R. R. Li Y. J. Chen T. T. (2019). Overexpression of the UGT76E12 gene modulates seed germination, growth, and response to NaCl, mannitol, and abscisic acid. *Biol. Plantarum* 63 328–334. 10.32615/bp.2019.038

[B31] Chen Z. Cuin T. A. Zhou M. Twomey A. Naidu B. P. Shabala S. (2007). Compatible solute accumulation and stress-mitigating effects in barley genotypes contrasting in their salt tolerance. *J. Exp. Bot.* 58 4245–4255. 10.1093/jxb/erm284 18182428

[B32] Cheng Z. Sun Y. Yang S. Zhi H. Yin T. Ma X. (2021). Establishing in planta haploid inducer line by edited SiMTL in foxtail millet (*Setaria italica*). *Plant Biotechnol. J.* 19:1089. 33749085 10.1111/pbi.13584 PMC8196629

[B33] Chivenge P. Mabhaudhi T. Modi A. T. Mafongoya P. (2015). The potential role of neglected and underutilised crop species as future crops under water scarce conditions in Sub-Saharan Africa. *Int. J. Environ. Res. Public Health* 12 5685–5711. 10.3390/ijerph120605685 26016431 PMC4483666

[B34] Christian M. Cermak T. Doyle E. L. Schmidt C. Zhang F. Hummel A. (2010). Targeting DNA double-strand breaks with TAL effector nucleases. *Genetics* 186 757–761. 10.1534/genetics.110.120717 20660643 PMC2942870

[B35] Crowe J. H. Crowe L. M. Carpenter J. F. Aurell Wistrom C. (1987). Stabilization of dry phospholipid bilayers and proteins by sugars. *Biochem. J.* 242 1–10. 10.1042/bj2420001 2954537 PMC1147656

[B36] Daneshmand F. Arvin M. J. Kalantari K. M. (2010). Physiological responses to NaCl stress in three wild species of potato in vitro. *Acta Physiol. Plantarum* 32 91–101. 10.1007/s11738-009-0384-2

[B37] Das P. Nutan K. K. Singla-Pareek S. L. Pareek A. (2015). Understanding salinity responses and adopting ‘omics-based ‘approaches to generate salinity tolerant cultivars of rice. *Front. Plant Sci.* 6:712. 10.3389/fpls.2015.00712 26442026 PMC4563168

[B38] Dell’Aversana E. Cirillo V. Van Oosten M. J. Di Stasio E. Saiano K. Woodrow P. (2021). Ascophyllum nodosum based extracts counteract salinity stress in tomato by remodeling leaf nitrogen metabolism. *Plants* 10:1044. 10.3390/plants10061044 34064272 PMC8224312

[B39] Deokar A. A. Tar’an B. (2016). Genome-wide analysis of the aquaporin gene family in chickpea (*Cicer arietinum* L.). *Front. Plant Sci.* 7:1802. 10.3389/fpls.2016.01802 27965700 PMC5126082

[B40] Desoky E. S. M. Merwad A. R. M. Rady M. M. (2018). Natural biostimulants improve saline soil characteristics and salt stressed-sorghum performance. *Commun. Soil Sci. Plant Anal.* 49 967–983. 10.1080/00103624.2018.1448861

[B41] Dolferus R. Ji X. Richards R. A. (2011). Abiotic stress and control of grain number in cereals. *Plant Sci.* 181 331–341. 10.1016/j.plantsci.2011.05.015 21889038

[B42] Dong O. X. Yu S. Jain R. Zhang N. Duong P. Q. Butler C. (2020). Marker-free carotenoid-enriched rice generated through targeted gene insertion using CRISPR-Cas9. *Nat. Commun.* 11:1178. 10.1038/s41467-020-14981-y 32132530 PMC7055238

[B43] Dugasa M. T. Cao F. Ibrahim W. Wu F. (2020). Difference physiological and biochemical characteristics in response to single and combined drought and salinity stresses between wheat genotypes differing in salt tolerance. *Physiol. Plant* 165 134–143. 10.1111/ppl.13032 29635753

[B44] Egamberdieva D. (2012). *Pseudomonas chlororaphis*: a salt-tolerant bacterial inoculant for plant growth stimulation under saline soil conditions. *Acta physiol. Plant.* 34 751–756. 10.1007/s11738-011-0875-9

[B45] Fan W. Zhang M. Zhang H. Zhang P. (2012). Improved tolerance to various abiotic stresses in transgenic sweet potato (Ipomoea batatas) expressing spinach betaine aldehyde dehydrogenase. *PLoS One* 7:e37344. 10.1371/journal.pone.0037344 22615986 PMC3353933

[B46] Feng Z. Zhang B. Ding W. Liu X. Yang D. L. Wei P. (2013). Efficient genome editing in plants using a CRISPR/Cas system. *Cell Res.* 23 1229–1232. 10.1038/cr.2013.114 23958582 PMC3790235

[B47] Ghosh S. Watson A. Gonzalez-Navarro O. E. Ramirez-Gonzalez R. H. Yanes L. Mendoza-Suárez M. (2018). Speed breeding in growth chambers and glasshouses for crop breeding and model plant research. *Nat. Protocols* 13 2944–2963. 10.1038/s41596-018-0072-z 30446746

[B48] Gupta B. Huang B. (2014). Mechanism of salinity tolerance in plants: physiological, biochemical, and molecular characterization. *Int. J. Genom.* 2014:701596. 10.1155/2014/701596 24804192 PMC3996477

[B49] Gupta B. K. Sahoo K. K. Ghosh A. Tripathi A. K. Anwar K. Das P. (2018). Manipulation of glyoxalase pathway confers tolerance to multiple stresses in rice. *Plant Cell Environ.* 41 1186–1200. 10.1111/pce.12968 28425127

[B50] Gupta S. M. Arora S. Mirza N. Pande A. Lata C. Puranik S. (2017). Finger millet: a “certain” crop for an “uncertain” future and a solution to food insecurity and hidden hunger under stressful environments. *Front. Plant Sci.* 8:643. 10.3389/fpls.2017.00643 28487720 PMC5404511

[B51] Hasthanasombut S. Ntui V. Supaibulwatana K. Mii M. Nakamura I. (2010). Expression of indica rice OsBADH1 gene under salinity stress in transgenic tobacco. *Plant Biotechnol. Rep.* 4 75–83. 10.1007/s11816-009-0123-6

[B52] Hatakeyama M. Aluri S. Balachadran M. T. Sivarajan S. R. Patrignani A. Grüter S. (2018). Multiple hybrid de novo genome assembly of finger millet, an orphan allotetraploid crop. *DNA Res.* 25 39–47. 10.1093/dnares/dsx036 28985356 PMC5824816

[B53] Hayat R. Ali S. Amara U. Khalid R. Ahmed I. (2010). Soil beneficial bacteria and their role in plant growth promotion: a review. *Annals Microbiol.* 60 579–598. 10.1007/s13213-010-0117-1

[B54] Hema R. Vemanna R. S. Sreeramulu S. Reddy C. P. Senthil-Kumar M. Udayakumar M. (2014). Stable expression of mtlD gene imparts multiple stress tolerance in finger millet. *PLoS One* 9:e99110. 10.1371/journal.pone.0099110 24922513 PMC4055669

[B55] Hittalmani S. Mahesh H. B. Shirke M. D. Biradar H. Uday G. Aruna Y. R. (2017). Genome and transcriptome sequence of finger millet (*Eleusine coracana* (L.) Gaertn.) provides insights into drought tolerance and nutraceutical properties. *BMC Genomics* 18:465. 10.1186/s12864-017-3850-z 28619070 PMC5472924

[B56] Horie T. Kaneko T. Sugimoto G. Sasano S. Panda S. K. Shibasaka M. (2011). Mechanisms of water transport mediated by PIP aquaporins and their regulation via phosphorylation events under salinity stress in barley roots. *Plant Cell Physiol.* 52 663–675. 10.1093/pcp/pcr027 21441236

[B57] Hossain M. S. Dietz K. J. (2016). Tuning of redox regulatory mechanisms, reactive oxygen species and redox homeostasis under salinity stress. *Front. Plant Sci.* 7:548. 10.3389/fpls.2016.00548 27242807 PMC4861717

[B58] Hu Y. Knapp S. Schmidhalter U. (2020). Advancing high-throughput phenotyping of wheat in early selection cycles. *Remote Sens.* 12:574. 10.3390/rs12030574

[B59] Ignacimuthu S. Ceasar S. A. (2012). Development of transgenic finger millet (*Eleusine coracana* (L.) Gaertn.) resistant to leaf blast disease. *J. Biosci.* 37 135–147. 10.1007/s12038-011-9178-y 22357211

[B60] Ishikawa T. Shabala S. (2019). Control of xylem Na+ loading and transport to the shoot in rice and barley as a determinant of differential salinity stress tolerance. *Physiol. Plant.* 165 619–631. 10.1111/ppl.12758 29761494

[B61] Islam M. T. Ckurshumova W. Fefer M. Liu J. Uddin W. Rosa C. (2021). A plant based modified biostimulant (copper chlorophyllin), mediates defense response in *Arabidopsis thaliana* under salinity stress. *Plants* 10:625. 10.3390/plants10040625 33806070 PMC8064443

[B62] James R. A. Blake C. Byrt C. S. Munns R. (2011). Major genes for Na+ exclusion, Nax1 and Nax2 (wheat HKT1; 4 and HKT1; 5), decrease Na+ accumulation in bread wheat leaves under saline and waterlogged conditions. *J. Exp. Bot.* 62 2939–2947. 10.1093/jxb/err003 21357768

[B63] Jayaprakash T. L. Ramamohan G. Krishnaprasad B. T. Prasad T. G. Mathew M. K. Udayakumar M. (1998). Genotypic variability in differential expression of lea2 and lea3 genes and proteins in response to salinity stress in finger millet (Eleusine coracana Gaertn) and rice (*Oryza sativa* L.) seedlings. *Annals Botany* 82 513–522. 10.1006/anbo.1998.0712

[B64] Jayasudha B. G. Sushma A. M. Prashantkumar H. S. Sashidhar V. R. (2014). An efficient in-vitro *Agrobacterium* mediated transformation protocol for raising salinity tolerant transgenic finger millet (*Eleusine coracana* (L.) Gaertn). *Plant Arch.* 14 823–829.

[B65] Jiang X. Leidi E. O. Pardo J. M. (2010). How do vacuolar NHX exchangers function in plant salt tolerance? *Plant Signal. Behav.* 5 792–795. 10.4161/psb.5.7.11767 20495345 PMC3014531

[B66] Kibria M. G. Hossain M. A. Murata Y. Hoque M. A. (2017). Antioxidant defense mechanisms of salinity tolerance in rice genotypes. *Rice Sci.* 24 155–162. 10.1016/j.rsci.2017.05.001

[B67] Kim D. Y. Jin J. Y. Alejandro S. Martinoia E. Lee Y. (2010). Overexpression of AtABCG36 improves drought and salt stress resistance in *Arabidopsis*. *Physiol. Plant.* 139 170–180. 10.1111/j.1399-3054.2010.01353.x 20088904

[B68] Kim M. J. Kim H. J. Pak J. H. Cho H. S. Choi H. K. Jung H. W. (2017). Overexpression of AtSZF2 from Arabidopsis showed enhanced tolerance to salt stress in soybean. *Plant Breed. Biotechnol.* 5 1–15. 10.9787/PBB.2017.5.1.1

[B69] Kothari S. L. Agarwal K. Kumar S. (2004). Inorganic nutrient manipulation for highly improved in vitro plant regeneration in finger millet-Eleusine coracana (L.) Gaertn. *Vitro Cell. Dev. Biology-Plant* 40 515–519. 10.1079/IVP2004564

[B70] Krishnamurthy L. Upadhyaya H. D. Purushothaman R. Gowda C. L. L. Kashiwagi J. Dwivedi S. L. (2014). The extent of variation in salinity tolerance of the minicore collection of finger millet (*Eleusine coracana* L. Gaertn.) germplasm. *Plant Sci.* 227 51–59. 10.1016/j.plantsci.2014.07.001 25219306

[B71] Kumar S. Kalita A. Srivastava R. Sahoo L. (2017). Co-expression of *Arabidopsis* NHX1 and bar improves the tolerance to salinity, oxidative stress, and herbicide in transgenic mungbean. *Front. Plant Sci.* 8:1896. 10.3389/fpls.2017.01896 29163616 PMC5673651

[B72] Kumar V. Khare T. (2016). Differential growth and yield responses of salt-tolerant and susceptible rice cultivars to individual (Na+ and Cl-) and additive stress effects of NaCl. *Acta Physiol. Plant.* 38 1–9. 10.1007/s11738-016-2191-x

[B73] Leonforte A. Forster J. W. Redden R. J. Nicolas M. E. Salisbury P. A. (2013). Sources of high tolerance to salinity in pea (*Pisum sativum* L.). *Euphytica* 189 203–216. 10.1007/s10681-012-0771-4

[B74] Li J. F. Norville J. E. Aach J. McCormack M. Zhang D. Bush J. (2013). Multiplex and homologous recombination-mediated genome editing in Arabidopsis and *Nicotiana benthamiana* using guide RNA and Cas9. *Nat. Biotechnol.* 31 688–691. 10.1038/nbt.2654 23929339 PMC4078740

[B75] Liang Z. Chen K. Li T. Zhang Y. Wang Y. Zhao Q. (2017). Efficient DNA-free genome editing of bread wheat using CRISPR/Cas9 ribonucleoprotein complexes. *Nat. Commun.* 8:14261. 10.1038/ncomms14261 28098143 PMC5253684

[B76] Liu G. Li J. Godwin I. D. (2019). “Genome editing by CRISPR/Cas9 in sorghum through biolistic bombardment,” in *Sorghum*, eds Zhao Z. Y. Dahlberg J. (New York, NY: Humana Press).

[B77] Liu X. Yang W. Mu B. Li S. Li Y. Zhou X. (2018). Engineering of ‘Purple Embryo Maize’ with a multigene expression system derived from a bidirectional promoter and self-cleaving 2A peptides. *Plant Biotechnol. J.* 16 1107–1109. 10.1111/pbi.12883 29337409 PMC5978582

[B78] Liu Y. Yu L. Qu Y. Chen J. Liu X. Hong H. (2016). GmSALT3, which confers improved soybean salt tolerance in the field, increases leaf Cl-exclusion prior to Na+ exclusion but does not improve early vigor under salinity. *Front. Plant Sci.* 7:1485. 10.3389/fpls.2016.01485 27746805 PMC5043451

[B79] Ma D. M. Xu W. R. Li H. W. Jin F. X. Guo L. N. Wang J. (2014). Co-expression of the Arabidopsis SOS genes enhances salt tolerance in transgenic tall fescue (*Festuca arundinacea* Schreb.). *Protoplasma* 51 219–231. 10.1007/s00709-013-0540-9 24022678 PMC3893463

[B80] Mahadik S. Kumudini B. S. (2020). Enhancement of salinity stress tolerance and plant growth in finger millet using fluorescent Pseudomonads. *Rhizosphere* 15:100226. 10.1016/j.rhisph.2020.100226

[B81] Mahalakshmi S. Christopher G. S. B. Reddy T. P. Rao K. V. Reddy V. D. (2006). Isolation of a cDNA clone (PcSrp) encoding serine-rich-protein from Porteresia coarctata T. and its expression in yeast and finger millet (*Eleusine coracana* L.) affording salt tolerance. *Planta* 224 347–359. 10.1007/s00425-005-0218-4 16450172

[B82] Manavalan L. P. Guttikonda S. K. Phan Tran L. S. Nguyen H. T. (2009). Physiological and molecular approaches to improve drought resistance in soybean. *Plant Cell Physiol.* 50 1260–1276. 10.1093/pcp/pcp082 19546148

[B83] Mariani L. Ferrante A. (2017). Agronomic management for enhancing plant tolerance to abiotic stresses-drought, salinity, hypoxia, and lodging. *Horticulturae* 3:52. 10.3390/horticulturae3040052

[B84] Mayak S. Tirosh T. Glick B. R. (2004). Plant growth-promoting bacteria confer resistance in tomato plants to salt stress. *Plant Physiol. Biochem.* 42 565–572. 10.1016/j.plaphy.2004.05.009 15246071

[B85] Mbarki S. Sytar O. Zivcak M. Abdelly C. Cerda A. Brestic M. (2018). Anthocyanins of coloured wheat genotypes in specific response to SalStress. *Molecules* 23:1518. 10.3390/molecules23071518 29937495 PMC6100425

[B86] Miladinovic D. Antunes D. Yildirim K. Bakhsh A. Cvejić S. Kondić-Špika A. (2021). Targeted plant improvement through genome editing: from laboratory to field. *Plant Cell Rep.* 40 935–951. 10.1007/s00299-020-02655-4 33475781 PMC8184711

[B87] Misra N. Gupta A. K. (2005). Effect of salt stress on proline metabolism in two high yielding genotypes of green gram. *Plant Sci.* 169 331–339. 10.1016/j.plantsci.2005.02.013

[B88] Mohammadi K. Movahedi A. Sadat Maleki S. Sun W. Zhang J. Yaghuti A. A. Z. (2018). Functional analysis of overexpressed PtDRS1 involved in abiotic stresses enhances growth in transgenic poplar. *Plant Physiol. Biochem.* 126 22–31. 10.1016/j.plaphy.2018.01.023 29494985

[B89] Mukami A. Ngetich A. Mweu C. Muthangya M. Oduor R. O. Ngugi M. (2018). Rapid and efficient plant regeneration from shoot apical meristems of finger millet [*Eleusine coracana* (L.) Gaertn.] via direct organogenesis. *African J. Biotechnol.* 17 898–905. 10.5897/AJB2018.16562

[B90] Mukami A. Ng’etich A. Syombua E. Oduor R. Mbinda W. (2020). Varietal differences in physiological and biochemical responses to salinity stress in six finger millet plants. *Physiol. Mol. Biol. Plants* 26 1569–1582. 10.1007/s12298-020-00853-8 32801487 PMC7415052

[B91] Munns R. (2002). Comparative physiology of salt and water stress. *Plant Cell Environ.* 25 239–250. 10.1046/j.0016-8025.2001.00808.x 11841667

[B92] Nekrasov V. Staskawicz B. Weigel D. Jones J. D. Kamoun S. (2013). Targeted mutagenesis in the model plant *Nicotiana benthamiana* using Cas9 RNA-guided endonuclease. *Nat. Biotechnol.* 31 691–693. 10.1038/nbt.2655 23929340

[B93] Ngetich A. Mweu C. Ngugi M. Mukami A. Ojulong H. Mbinda W. (2018). Efficient plant regeneration protocol for finger millet [*Eleusine coracana* (L.) Gaertn.] via somatic embryogenesis. *African J. Biotechnol.* 17 660–667. 10.5897/AJB2018.16452

[B94] Nia S. H. Zarea M. J. Rejali F. Varma A. (2012). Yield and yield components of wheat as affected by salinity and inoculation with *Azospirillum* strains from saline or non-saline soil. *J. Saudi Soc. Agricultural Sci.* 11 113–121. 10.1016/j.jssas.2012.02.001

[B95] Nishizawa-Yokoi A. Yabuta Y. Shigeoka S. (2008). The contribution of carbohydrates including raffinose family oligosaccharides and sugar alcohols to protection of plant cells from oxidative damage. *Plant Signal. Behav.* 3 1016–1018. 10.4161/psb.6738 19704439 PMC2633762

[B96] Noori F. Etesami H. Noori S. Forouzan E. Jouzani G. S. Malboobi M. A. (2021). Whole genome sequence of *Pantoea agglomerans* ANP8, a salinity and drought stress–resistant bacterium isolated from alfalfa (*Medicago sativa* L.) root nodules. *Biotechnol. Rep.* 29:e00600. 10.1016/j.btre.2021.e00600 33643858 PMC7893418

[B97] Onyango A. O. (2016). Finger millet: food security crop in the arid and semi-arid lands (ASALs) of Kenya. *World Environ.* 6 62–70. 10.5923/j.env.20160602.03 22499009

[B98] Ozturk L. Demir Y. Unlukara A. Karatas I. Kurunc A. Duzdemir O. (2012). Effects of long-term salt stress on antioxidant system, chlorophyll and proline contents in pea leaves. *Romanian Biotechnol. Lett.* 17 7227–7236.

[B99] Pehlivan N. Sun L. Jarrett P. Yang X. Mishra N. Chen L. (2016). Co-overexpressing a plasma membrane and a vacuolar membrane sodium/proton antiporter significantly improves salt tolerance in transgenic *Arabidopsis* plants. *Plant Cell Physiol.* 57 1069–1084. 10.1093/pcp/pcw055 26985021 PMC4867051

[B100] Peterson B. A. Haak D. C. Nishimura M. T. Teixeira P. J. James S. R. Dangl J. L. (2016). Genome-wide assessment of efficiency and specificity in CRISPR/Cas9 mediated multiple site targeting in *Arabidopsis*. *PLoS One* 11:e0162169. 10.1371/journal.pone.0162169 27622539 PMC5021288

[B101] Preston B. (2013). Synthetic biology as red herring. *Stud. History Philos. Sci. Part C: Stud. History Philos. Biol. Biomed. Sci.* 44 649–659. 10.1016/j.shpsc.2013.05.012 23816689

[B102] Puchta H. Dujon B. Hohn B. (1993). Homologous recombination in plant cells is enhanced by in vivo induction of double strand breaks into DNA by a site-specific endonuclease. *Nucleic Acids Res.* 21 5034–5040. 10.1093/nar/21.22.5034 8255757 PMC310614

[B103] Rafiq K. Akram M. S. Shahid M. Qaisar U. Rashid N. (2020). Enhancement of salt tolerance in maize (*Zea mays* L.) using locally isolated *Bacillus sp*. SR-2-1/1. *Biologia* 75 1425–1436. 10.2478/s11756-020-00435-9

[B104] Rahman H. Jagadeeshselvam N. Valarmathi R. Sachin B. Sasikala R. Senthil N. (2014). Transcriptome analysis of salinity responsiveness in contrasting genotypes of finger millet (*Eleusine coracana* L.) through RNA-sequencing. *Plant Mol. Biol.* 85 485–503. 10.1007/s11103-014-0199-4 24838653

[B105] Rahman H. Ramanathan V. Nallathambi J. Duraialagaraja S. Muthurajan R. (2016). Over-expression of a NAC 67 transcription factor from finger millet (*Eleusine coracana* L.) confers tolerance against salinity and drought stress in rice. *BMC Biotechnol.* 16:7–20. 10.1186/s12896-016-0261-1 27213684 PMC4896240

[B106] Rahnama A. James R. A. Poustini K. Munns R. (2010). Stomatal conductance as a screen for osmotic stress tolerance in durum wheat growing in saline soil. *Funct. Plant Biol.* 37 255–263. 10.1071/FP09148

[B107] Ramadoss D. Lakkineni V. K. Bose P. Ali S. Annapurna K. (2013). Mitigation of salt stress in wheat seedlings by halotolerant bacteria isolated from saline habitats. *SpringerPlus* 2:6. 10.1186/2193-1801-2-6 23449812 PMC3579424

[B108] Ramakrishna C. Singh S. Raghavendrarao S. Padaria J. C. Mohanty S. Sharma T. R. (2018). The membrane tethered transcription factor EcbZIP17 from finger millet promotes plant growth and enhances tolerance to abiotic stresses. *Sci. Rep.* 8:2148. 10.1038/s41598-018-19766-4 29391403 PMC5794737

[B109] Rana M. M. Takamatsu T. Baslam M. Kaneko K. Itoh K. Harada N. (2019). Salt tolerance improvement in rice through efficient SNP marker-assisted selection coupled with speed-breeding. *Int. J. Mol. Sci.* 20:2585. 10.3390/ijms20102585 31130712 PMC6567206

[B110] Rao E. S. Kadirvel P. Symonds R. C. Ebert A. W. (2013). Relationship between survival and yield related traits in *Solanum pimpinellifolium* under salt stress. *Euphytica* 190 215–228. 10.1007/s10681-012-0801-2

[B111] Rasool S. Ahmad A. Siddiqi T. O. Ahmad P. (2013). Changes in growth, lipid peroxidation and some key antioxidant enzymes in chickpea genotypes under salt stress. *Acta Physiol. Plant.* 35 1039–1050. 10.1007/s11738-012-1142-4

[B112] Razali R. Bougouffa S. Morton M. J. Lightfoot D. J. Alam I. Essack M. (2018). The genome sequence of the wild tomato *Solanum pimpinellifolium* provides insights into salinity tolerance. *Front. Plant Sci.* 9:1402. 10.3389/fpls.2018.01402 30349549 PMC6186997

[B113] Resende R. T. Piepho H. P. Rosa G. J. Silva-Junior O. B. e Silva F. F. de Resende M. D. V. (2021). Enviromics in breeding: applications and perspectives on envirotypic-assisted selection. *Theoretical Appl. Genetics* 134 95–112. 10.1007/s00122-020-03684-z 32964262

[B114] Roell M. S. Zurbriggen M. D. (2020). The impact of synthetic biology for future agriculture and nutrition. *Curr. Opin. Biotechnol.* 61 102–109. 10.1016/j.copbio.2019.10.004 31812911

[B115] Sagar M. Chervin C. Mila I. Hao Y. Roustan J. P. Benichou M. (2013). SlARF4, an auxin response factor involved in the control of sugar metabolism during tomato fruit development. *Plant Physiol.* 161 1362–1374. 10.1104/pp.113.213843 23341361 PMC3585602

[B116] Sakamma S. Umesh K. B. Girish M. R. Ravi S. C. Satishkumar M. Bellundagi V. (2018). Finger millet (*Eleusine coracana* L. Gaertn.) production system: status, potential, constraints and implications for improving small farmer’s welfare. *J. Agricultural Sci.* 10 162–179. 10.5539/jas.v10n1p162

[B117] Sánchez-León S. Gil-Humanes J. Ozuna C. V. Giménez M. J. Sousa C. Voytas D. F. (2018). Low-gluten, nontransgenic wheat engineered with CRISPR/Cas9. *Plant Biotechnol. J.* 16 902–910. 10.1111/pbi.12837 28921815 PMC5867031

[B118] Sarabi B. Bolandnazar S. Ghaderi N. Ghashghaie J. (2017). Genotypic differences in physiological and biochemical responses to salt stress in melon (*Cucumis melo* L.) plants. Prospects for selection of salt tolerant landraces. *Plant Physiol. Biochem.* 119 294–311. 10.1016/j.plaphy.2017.09.006 28938176

[B119] Satish L. Ceasar S. A. Ramesh M. (2017). Improved Agrobacterium-mediated transformation and direct plant regeneration in four cultivars of finger millet (*Eleusine coracana* (L.) Gaertn.). *Plant Cell Tissue Organ Culture* 131 547–565. 10.1007/s11240-017-1305-5

[B120] Schmidt C. Pacher M. Puchta H. (2019). “DNA break repair in plants and its application for genome engineering,” in *Transgenic Plants. Methods in Molecular Biology*, eds Kumar S. Barone P. Smith M. (New York, NY: Humana Press), 10.1007/978-1-4939-8778-8_17 30415341

[B121] Shafi A. Pal A. K. Sharma V. Kalia S. Kumar S. Ahuja P. S. (2017). Transgenic potato plants overexpressing SOD and APX exhibit enhanced lignification and starch biosynthesis with improved salt stress tolerance. *Plant Mol. Biol. Reporter* 35 504–518. 10.1007/s11105-017-1041-3

[B122] Shah A. N. Tanveer M. Abbas A. Fahad S. Baloch M. S. Ahmad M. I. (2021). Targeting salt stress coping mechanisms for stress tolerance in *Brassica*: a research perspective. *Plant Physiol. Biochem.* 158 53–64. 10.1016/j.plaphy.2020.11.044 33296846

[B123] Shah Z. H. Rehman H. M. Akhtar T. Daur I. Nawaz M. A. Ahmad M. Q. (2017). Redox and ionic homeostasis regulations against oxidative, salinity and drought stress in wheat (a systems biology approach). *Front. Genetics* 8:141. 10.3389/fgene.2017.00141 29089961 PMC5651134

[B124] Shahzad B. Rehman A. Tanveer M. Wang L. Park S. K. Ali A. (2021). Salt stress in *Brassica*: effects, tolerance mechanisms, and management. *J. Plant Growth Regulat.* 1–15. 10.1007/s00344-021-10338-x

[B125] Shan Q. Wang Y. Li J. Zhang Y. Chen K. Liang Z. (2013). Targeted genome modification of crop plants using a CRISPR-Cas system. *Nat. Biotechnol.* 31 686–688. 10.1038/nbt.2650 23929338

[B126] Sharma I. Bhardwaj R. Pati P. K. (2013). Stress modulation response of 24-epibrassinolide against imidacloprid in an elite indica rice variety Pusa Basmati-1. *Pesticide Biochem. Physiol.* 105 144–153. 10.1016/j.pestbp.2013.01.004

[B127] Singla-Pareek S. L. Reddy M. K. Sopory S. K. (2003). Genetic engineering of the glyoxalase pathway in tobacco leads to enhanced salinity tolerance. *Proc. Natl. Acad. Sci. U S A.* 100 14672–14677. 10.1073/pnas.2034667100 14638937 PMC299757

[B128] Soliman W. S. Sugiyama S. I. Abbas A. M. (2018). Contribution of avoidance and tolerance strategies towards salinity stress resistance in eight C3 turfgrass species. *Horticulture Environ. Biotechnol.* 59 29–36. 10.1007/s13580-018-0004-4

[B129] Sripinyowanich S. Klomsakul P. Boonburapong B. Bangyeekhun T. Asami T. Gu H. (2013). Exogenous ABA induces salt tolerance in indica rice (*Oryza sativa* L.): the role of OsP5CS1 and OsP5CR gene expression during salt stress. *Environ. Exp. Botany* 86 94–105. 10.1016/j.envexpbot.2010.01.009

[B130] Sun H. Li L. Lou Y. Zhao H. Yang Y. Wang S. (2017). The bamboo aquaporin gene PeTIP4; 1–1 confers drought and salinity tolerance in transgenic *Arabidopsis*. *Plant Cell Rep.* 36 597–609. 10.1007/s00299-017-2106-2103 28168515

[B131] Taïbi K. Taïbi F. Abderrahim L. A. Ennajah A. Belkhodja M. Mulet J. M. (2016). Effect of salt stress on growth, chlorophyll content, lipid peroxidation and antioxidant defence systems in *Phaseolus vulgaris* L. *South African J. Botany* 105 306–312. 10.1016/j.sajb.2016.03.011

[B132] Tang L. Cai H. Ji W. Luo X. Wang Z. Wu J. (2013). Overexpression of GsZFP1 enhances salt and drought tolerance in transgenic alfalfa (*Medicago sativa* L.). *Plant Physiol. Biochem.* 71 22–30. 10.1016/j.plaphy.2013.06.024 23867600

[B133] Tang L. Mao B. Li Y. Lv Q. Zhang L. Chen C. (2017). Knockout of OsNramp5 using the CRISPR/Cas9 system produces low Cd-accumulating indica rice without compromising yield. *Sci. Rep.* 7:14438. 10.1038/s41598-017-14832-9 29089547 PMC5663754

[B134] Tekaligne T. M. Woldu A. R. Tsigie Y. A. (2015). Bioethanol production from finger millet (*Eleusine coracana*) straw. *Ethiopian J. Sci. Technol.* 8:1. 10.4314/ejst.v8i1.1

[B135] Thilakarathna M. S. Raizada M. N. (2015). A review of nutrient management studies involving finger millet in the semi-arid tropics of Asia and Africa. *Agronomy* 5 262–290. 10.3390/agronomy5030262

[B136] Tiwari S. Singh P. Tiwari R. Meena K. K. Yandigeri M. Singh D. P. (2011). Salt-tolerant rhizobacteria-mediated induced tolerance in wheat (*Triticum aestivum*) and chemical diversity in rhizosphere enhance plant growth. *Biol. Fertility Soils* 47:907. 10.1007/s00374-011-0598-5

[B137] Upadhyay S. K. Singh J. S. Saxena A. K. Singh D. P. (2012). Impact of PGPR inoculation on growth and antioxidant status of wheat under saline conditions. *Plant Biol.* 14 605–611. 10.1111/j.1438-8677.2011.00533.x 22136617

[B138] Vadez V. Rashmi M. Sindhu K. Muralidharan M. Pushpavalli R. Turner N. C. (2012). Large number of flowers and tertiary branches, and higher reproductive success increase yields under salt stress in chickpea. *Eur. J. Agronomy* 41 42–51. 10.1016/j.eja.2012.03.008

[B139] Van Oosten M. J. Pepe O. De Pascale S. Silletti S. Maggio A. (2017). The role of biostimulants and bioeffectors as alleviators of abiotic stress in crop plants. *Chem. Biol. Technol. n Agriculture* 4:5. 10.1186/s40538-017-0089-5

[B140] Viveros M. F. Á Inostroza-Blancheteau C. Timmermann T. González M. Arce-Johnson P. (2013). Overexpression of GlyI and GlyII genes in transgenic tomato (*Solanum lycopersicum* Mill.) plants confers salt tolerance by decreasing oxidative stress. *Mol. Biol. Rep.* 40 3281–3290. 10.1007/s11033-012-2403-4 23283739

[B141] Wang L. Chen L. Li R. Zhao R. Yang M. Sheng J. (2017). Reduced drought tolerance by CRISPR/Cas9-mediated SlMAPK3 mutagenesis in tomato plants. *J. Agric. Food Chem.* 65 8674–8682. 10.1021/acs.jafc.7b02745 28873302

[B142] Wang M. Mao Y. Lu Y. Tao X. Zhu J. K. (2017). Multiplex gene editing in rice using the CRISPR-Cpf1 system. *Mol. Plant* 10 1011–1013. 10.1016/j.molp.2017.03.001 28315752

[B143] Wang Q. Wu C. Xie B. Liu Y. Cui J. Chen G. (2012). Model analysing the antioxidant responses of leaves and roots of switchgrass to NaCl-salinity stress. *Plant Physiol. Biochem.* 58 288–296. 10.1016/j.plaphy.2012.06.021 22871483

[B144] Wang W. Wu Z. He Y. Huang Y. Li X. Ye B. C. (2018). Plant growth promotion and alleviation of salinity stress in *Capsicum annuum* L. by Bacillus isolated from saline soil in Xinjiang. *Ecotoxicol. Environ. Safety* 164 520–529. 10.1016/j.ecoenv.2018.08.070 30149350

[B145] Wang Y. Jiang L. Chen J. Tao L. An Y. Cai H. (2018). Overexpression of the alfalfa WRKY11 gene enhances salt tolerance in soybean. *PLoS One* 13:e0192382. 10.1371/journal.pone.0192382 29466387 PMC5821330

[B146] Watson A. Ghosh S. Williams M. J. Cuddy W. S. Simmonds J. Rey M. D. (2018). Speed breeding is a powerful tool to accelerate crop research and breeding. *Nat. Plants* 4 23–29. 10.1038/s41477-017-0083-8 29292376

[B147] Weisany W. Sohrabi Y. Heidari G. Siosemardeh A. Ghassemi-Golezani K. (2012). Changes in antioxidant enzymes activity and plant performance by salinity stress and zinc application in soybean (‘*Glycine max*’ L.). *Plant Omics* 5 60–67. 10.3316/informit.182984019960534 25677225

[B148] Werner C. R. Gaynor R. C. Gorjanc G. Hickey J. M. Kox T. Abbadi A. (2020). How population structure impacts genomic selection accuracy in cross-validation: implications for practical breeding. *Front. Plant Sci.* 11:2028. 10.3389/fpls.2020.592977 33391305 PMC7772221

[B149] Wright D. A. Townsend J. A. Winfrey R. J. Jr. Irwin P. A. Rajagopal J. Lonosky P. M. (2005). High-frequency homologous recombination in plants mediated by zinc-finger nucleases. *Plant J.* 44 693–705. 10.1111/j.1365-313X.2005.02551.x 16262717

[B150] Wurtzel E. T. Vickers C. E. Hanson A. D. Millar A. H. Cooper M. Voss-Fels K. P. (2019). Revolutionizing agriculture with synthetic biology. *Nat. Plants* 5 1207–1210. 10.1038/s41477-019-0539-0 31740769

[B151] Yan H. Li Q. Park S. C. Wang X. Liu Y. J. Zhang Y. G. (2016). Overexpression of CuZnSOD and APX enhance salt stress tolerance in sweet potato. *Plant Physiol. Biochem.* 109 20–27. 10.1016/j.plaphy.2016.09.003 27620271

[B152] Yao L. Wu Z. Zheng Y. Kaleem I. Li C. (2010). Growth promotion and protection against salt stress by *Pseudomonas putida* Rs-198 on cotton. *Eur. J. Soil Biol.* 46 49–54. 10.1016/j.ejsobi.2009.11.002

[B153] Yepes-Molina L. Bárzana G. Carvajal M. (2020). Controversial regulation of gene expression and protein transduction of aquaporins under drought and salinity stress. *Plants* 9:1662. 10.3390/plants9121662 33261103 PMC7761296

[B154] Yu J. Q. Wang J. H. Sun C. H. Zhang Q. Y. Hu D. G. Hao Y. J. (2018). Ectopic expression of the apple nucleus-encoded thylakoid protein MdY3IP1 triggers early-flowering and enhanced salt-tolerance in *Arabidopsis thaliana*. *BMC Plant Biol.* 18:18. 10.1186/s12870-018-1232-6 29352810 PMC5775602

[B155] Zhang A. Liu Y. Wang F. Li T. Chen Z. Kong D. (2019). Enhanced rice salinity tolerance via CRISPR/Cas9-targeted mutagenesis of the OsRR22 gene. *Mol. Breed.* 39:47. 10.1007/s11032-019-0954-y 32803201 PMC7413041

[B156] Zhang W. J. Wang T. (2015). Enhanced salt tolerance of alfalfa (*Medicago sativa*) by rstB gene transformation. *Plant Sci.* 234 110–118. 10.1016/j.plantsci.2014.11.016 25804814

[B157] Zhang W. Zhi H. Tang S. Zhang H. Sui Y. Jia G. (2021). Identification of no pollen 1 provides a candidate gene for heterosis utilization in foxtail millet (*Setaria italica* L.). *Crop J.* 10.1016/j.cj.2021.03.018 (in press).

[B158] Zheng L. Chen S. Xie L. Lu Z. Liu M. Han X. (2018). Overexpression of cysteine protease gene from Salix matsudana enhances salt tolerance in transgenic *Arabidopsis*. *Environ. Exp. Bot.* 147 53–62. 10.1016/j.envexpbot.2017.11.008

[B159] Zou J. Liu C. Liu A. Zou D. Chen X. (2012). Overexpression of OsHsp17. 0 and OsHsp23. 7 enhances drought and salt tolerance in rice. *J. Plant Physiol.* 169 628–635. 10.1016/j.jplph.2011.12.014 22321692

